# Une urgence médico-chirurgicale rare: l’abcès épidural rachidien (à propos de 03 cas)

**DOI:** 10.11604/pamj.2017.26.145.9156

**Published:** 2017-03-14

**Authors:** Abderrazzak El Saqui, Mohamed Aggouri, Mohamed Benzagmout, Khalid Chakour, Mohamed El Faiz Chaoui

**Affiliations:** 1 Service Neurochirurgie, CHU Hassan II, Fès, Maroc

**Keywords:** Abcès épidural, infection, IRM, chirurgie, Epidural abscess, infection, MRI, surgery

## Abstract

Les infections de l’espace épidural sont de mieux en mieux connues grâce au développement de la neurochirurgie, notamment l’IRM. Les abcès épiduraux rachidiens représentent une pathologie rare mais éminemment grave sur le plan fonctionnel, avec un risque vital potentiel. Nous rapportons trois cas d’abcès épidural rachidien tous diagnostiqués chez des patients de sexe masculin, le premier âgé de 52 ans le deuxième de 57 ans et le troisième de 63ans. Deux patients ont été admis aux urgences neurochirurgicales pour un tableau de compression médullaire lente évoluant dans un contexte infectieux, et le dernier se plaignait d’une sciatique S1 droite rebelle au traitement avec des fuites urinaires. Aucune porte d’entrée n’a été identifiée dans le bilan initial. Tous les patients ont été opérés par voie d’abord postérieure avec décompression médullaire/radiculaire et évacuation de l’abcès épidural. L’étude bactériologique a trouvé un germe pyogène justifiant une antibiothérapie adaptée dans les trois cas. L’évolution a été favorable dans deux cas. Par contre un patient est décédé trois jours en post-opératoire par un sepsis sévère.

## Abstract (en)

The awareness about infections in the epidural space is increasing thanks to the development of neurosurgery, including MRI. Spinal epidural abscess is a rare pathology but extremely serious from a functional point of view and potentially life threatening. We report three cases of male patients (the first one aged 52 years, the second 57 years and the third 63 years) with diagnosed spinal epidural abscess. Two patients were admitted to the Neurosurgical Emergencies with slow progressive spinal cord compression evolving in the context of infection. The last patient complained of S1 sciatica pain in his right leg resistant to treatment associated with urinary incontinence. Entrance door of the infection wasn’t identified during the initial assessment. All patients underwent spinal cord/radicular decompression surgery and evacuation of the epidural abscess via posterior approach. Bacteriological examination showed pyogenic germ justifying adequate prescription of antibiotic therapy in the three cases. The evolution was favorable in two cases. However one patient died three days after surgery due to severe sepsis.

## Introduction

Les abcès épiduraux constituent une véritable urgence diagnostique car la prise en charge thérapeutique doit être rapide et efficace pour éviter de graves séquelles neurologiques. Les signes cliniques et biologiques sont variables et souvent trompeurs. L´imagerie, et en particulier l´imagerie par résonance magnétique, à un rôle décisif pour le diagnostic. Le traitement médical adéquat instauré précocement est la clé du traitement; cependant, certains cas nécessitent le recours à une décompression chirurgicale. L’évolution est fonction du tableau clinique initial et de la rapidité de la prise en charge.

## Patient et observation

Nous rapportons ci-après l’expérience du service de Neurochirurgie CHU Hassan II- Fès concernant la prise en charge de l’abcès épidural rachidien à travers l’analyse rétrospective de trois cas.

### Observation 1

**Figure 1:**
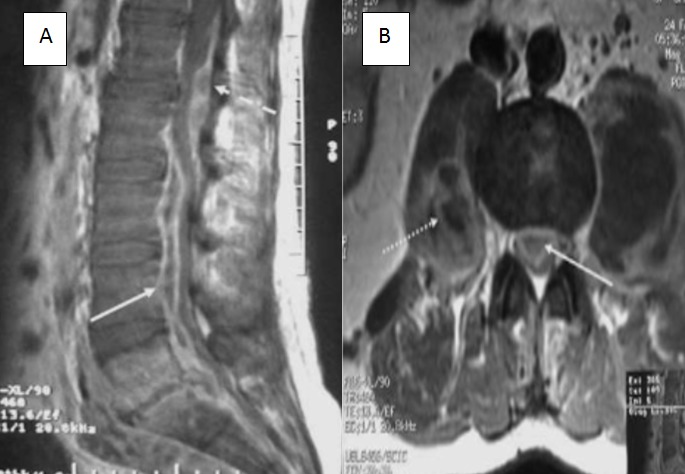
A) IRM médullaire en coupe sagittale, séquence pondérée T1 avec injection de gadolinium montrant une collection épidurale antérieure s’étendant de L2 à L5 (flèche pleine). Noter l’existence d’une collection épidurale postérieure associée en regard de l’espace intervertébral L1-L2 (flèche en pointillés); B) IRM médullaire en coupe axiale, passant par L3, montrant une collection épidurale antérieure exerçant une forte compression sur le fourreau dural (flèche pleine), associée à des abcès de psoas (flèche en pointillés)

Monsieur K.D est un patient âgé de 52 ans, sans antécédents pathologiques notables, admis aux urgences neurochirurgicales en Février 2004 pour un syndrome de la queue de cheval incomplet évoluant depuis deux mois, dans un contexte de fièvre non chiffrée et de conservation de l’état général. L’examen neurologique initial avait révélé une paraparésie grade C de Frankel sans déficit sensitif associé. L’examen général a trouvé une fébricule à 38°C. Cependant, aucune porte d’entrée n’a été identifiée dans le bilan initial. Une IRM rachidienne avec injection de gadolinium a été alors réalisée en urgence et a montré l’existence d’une collection épidurale antérieure, apparaissant en hyposignal T1, hypersignal T2, prenant le gadolinium en périphérie et siégeant en regard des quatre dernières vertèbres lombaires. Il a été également noté l’existence de collections hypointenses prenant le contraste en périphérie au niveau des deux muscles psoas (Figure 1). Le diagnostic d’abcès épidural rachidien associé à des abcès de psoas a été évoqué et le bilan biologique a montré un syndrome inflammatoire avec une vitesse de sédimentation (VS) à 110 mm à la première heure et une protéine C réactive (CRP) à 80 mg/l. L’intradermoréaction à la tuberculine était négative. L’indication chirurgicale a été retenue vu l’importance du déficit neurologique et le volume assez important de l’abcès épidural sur l’IRM. C’est ainsi que le patient a été opéré par voie postérieure avec réalisation d’une laminectomie étendue de L2 à L5, aspiration de l’abcès épidural après écartement du fourreau dural. La collection épidurale était liquéfiée, de couleur jaune verdâtre et d’odeur fétide. L’étude bactériologique a identifié un germe pyogène (staphylocoque aureus) ayant justifié l’instauration d’une double antibiothérapie par voie parentérale associant une amoxicilline protégée (Augmentin. 6g/j) et un aminoside (Gentamycine. 160mg/j). Ce traitement a été poursuivi pendant cinq jours pour l’aminoside et trois semaines pour l’amoxicilline protégée, puis relayé par une antibiothérapie orale à base de Ciprofloxacine (1,5g/jour) pendant un mois. L’évolution clinique a été favorable avec une récupération quasi-totale du déficit moteur après trois mois de rééducation postopératoire.

### Observation 2

**Figure 2:**
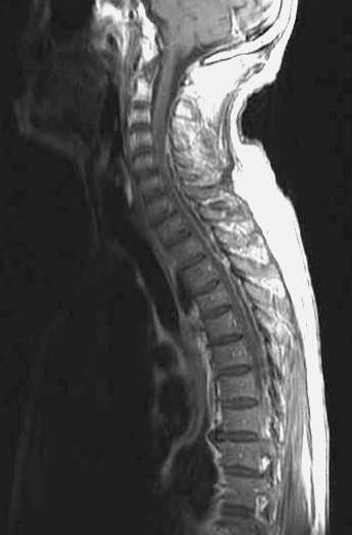
IRM médullaire cervico-thoracique en coupe sagittale, séquence pondérée T1 avec injection de produit de contraste, montrant une collection épidurale postérieure, étendue de C4 à T7, prenant le contraste en périphérie et refoulant la moelle

Monsieur M.G âgé de 62 ans, sans antécédents pathologiques particuliers, a été admis aux urgences neurochirurgicales pour une lourdeur des 4 membres d’aggravation rapidement progressive associée à des troubles génito-sphinctériens. L’interrogatoire du patient a retrouvé la notion de rachialgies dorsales hautes, de type inflammatoire, évoluant depuis deux mois dans un contexte fébrile. A l’admission, l’examen clinique a trouvé un patient ayant un bon état hémodynamique mais un état général altéré avec une fièvre à 38,5°C, un teint terreux, des plis de déshydratation et un début d’escarres au niveau sacré. L’examen neurologique a objectivé une tétraplégie spastique grade A de Frankel. L’IRM médullaire a mis en évidence une collection épidurale postérieure cervico-thoracique très étendue, de C4 à T7, en hyposignal T1, hypersignal T2, prenant le gadolinium en périphérie, fort évocatrice d’un abcès épidural rachidien (Figure 2). Par ailleurs, les segments osseux rachidiens et les parties molles de voisinage étaient sans anomalies, Le bilan biologique a noté une augmentation significative de la VS et de la CRP (VS à 90 mm à la première heure et une CRP à 260 mg/l). Par ailleurs, les sérologies virales, notamment l’HIV, et les hémocultures étaient négatives. Après des mesures de réanimation initiale ayant consisté en une bonne réhydratation du patient, ce dernier a été opéré par voie postérieure au travers d’une laminectomie étendue de C5 à T3. Un prélèvement du pus a été réalisé en per-opératoire avant de procéder à l’aspiration drainage de l’abcès épidural au moyen d’un drain aspiratif. Les prélèvements bactériologiques ont mis en évidence un staphylocoque méthicillino-résistant. Une double antibiothérapie a été instaurée à base de Vancomycine (Vancocine. 2g/jour) et de Fosfomycine (Fosfocine. en IV 12g/jour). En postopératoire immédiat, le statut neurologique du patient est resté inchangé. Par ailleurs, l’évolution était marquée par la survenue d’un choc septique réfractaire et le malade est décédé au service de réanimation trois jours plus tard dans un tableau de défaillance multi-viscérale.

### Observation 3

**Figure 3:**
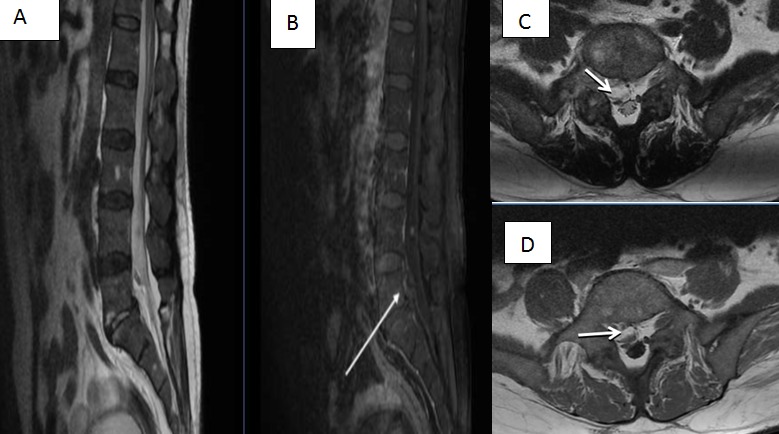
IRM rachidienne lombaire en coupes sagittales, séquences pondérées T1 avec Gadolinium (B) et T2 (A) montrant une collection épidurale antérieure prenant le contraste, rétrocorporéale L5; (C) et (D) Coupes axiales T2 du même patient montrant la collection épidurale antéro-latérale droite, se prolongeant au niveau foraminal, conflictuelle avec la racine L5 droite

Monsieur M .A est un patient âgé de 67 ans, suivi pour un diabète insulino-dépendant depuis une quinzaine d’années ; mal équilibré sous insuline rapide. Le patient accuse une lombosciatique droite, mal systématisée, rebelle au traitement symptomatique depuis deux mois, évoluant dans un contexte de fièvre non chiffrée et d’amaigrissement chiffré à 06 kg en deux mois. L’examen clinique à l’admission a trouvé un patient conscient, ayant un assez bon état général, subfébrile à 37,8°C, avec un bon état hémodynamique. L’examen neurologique était sans particularités et l’examen rachidien a objectivé une raideur rachidienne manifeste. Le signe de Lasègue était évalué à 40° à droite et 70° à gauche. L’IRM rachidienne lombaire avait révélé l’existence d’une collection épidurale antéro-latérale droite, se projetant en regard du disque intervertébral L5-S1 prenant le contraste, se prolongeant au niveau foraminale, conflictuelle avec la racine L5 droite (Figure 3). Le bilan biologique a montré une glycémie à jeûn à 2,5g/l avec une hémoglobine glyquée à 11%. La fonction rénale était correcte et le bilan inflammatoire perturbé (VS à 70 à la première heure et CRP à 96 mg/l). Le patient a été opéré par voie postérieure. En post opératoire immédiat, il a rapporté un soulagement net de la douleur radiculaire avec amélioration des signes infectieux. Au cours de la première semaine, l’étude bactériologique était négative. Le patient a été mis sous bi-antibiothérapie associant une Céphalosporine de 3ème génération et une vancomycine pendant deux mois avec une évolution favorable.

## Discussion

L’abcès épidural spinal (AES) a été décrit pour la 1^ère^ fois en 1761 par l’anatomiste italien Morgagni [1]. Collection septique de l’espace épidural, l’AES affecte préférentiellement les individus de sexe masculin (rapport M/F = 1/0,56) âgés de plus de 50 ans, mais peut être diagnostiqué à tout âge. Son incidence (0,2-2,0/10000 hospitalisations, soit moins de 1 cas/ million de personnes) est en augmentation, essentiellement dû au vieillissement de la population, à l’utilisation plus large de traitements immunosuppresseurs, de corticostéroides, et à la multiplication de gestes médicaux invasifs sur le rachis [2]. Les autres facteurs de risques sont les déficiences immunitaires congénitales ou acquises, le diabète, l’insuffisance rénale, la toxicomanie par voie intraveineuse, l’alcoolisme, les traumatismes récents, les infections intercurrentes avec bactériémie, le post-partum voire les anomalies congénitales occultes de fermeture de l’arc neural telle que la persistance d’un sinus dermique [3]. L’examen minutieux des téguments sur la ligne médiane à la recherche d’une anomalie dysraphique revêt donc une importance primordiale. Lorsqu’elle n’est pas instrumentale, la dissémination du germe à l’espace épidural se fait par voie hématogène (furoncle, infection pelvienne, pulmonaire, endocardite …) ou par continuité (AES compliquant une spondylodiscite). Par ordre de fréquence décroissant, c’est l’étage dorsal puis lombaire du rachis qui est principalement concerné. L’agent causal le plus fréquemment isolé est le staphylocoque doré (66-85%), suivi par les bactéries Gram négatif et la tuberculose. Une étiologie mycosique ou parasitaire a été rapportée chez quelques patients sévèrement immunodéprimés. La présentation clinique habituelle se caractérise par des douleurs (70-85%) accompagnée de fièvre (66%), une contracture musculaire paravertébrale (24-90%), un déficit neurologique sensitivo-moteur (39-50%) et des troubles sphinctériens (27-44%). En 1948, Heusner décrit pour la première fois l’évolution clinique de l’AES qu’il divise en 4 stades de gravité croissante: le 1er stade se caractérise par une douleur intense associée à une contracture locale et de la fièvre; le 2ème stade par une irritation spinale avec une positivité des signes de Lasègue, Kernig, Lhermitte et Brudzinski; le 3^e^ stade par un déficit neurologique sensitif et/ou sphinctérien; le 4^e^ stade par une atteinte motrice sévère progressant vers la paraplégie. Les examens de laboratoire démontrent un syndrome inflammatoire (vitesse de sédimentation et C-réactive-protéine augmentées, anémie microcytaire hypochrome régénérative) voire infectieux (hyperleucocytose avec déviation gauche à la formule sanguine complète).

Devant cette présentation clinique suggestive mais aspécifique, les investigations radiologiques sont indispensables à la confirmation du diagnostic, au bilan d’extension et à la recherche de lésions associées ou causale, telle que la spondylodiscite. L’IRM, avec injection intraveineuse de gadolinium, est la technique de référence pour l’analyse du canal rachidien et son contenu. Sa sensibilité dans cette indication est supérieure à 95%. Les performances du scanner natif sont insuffisantes pour ce diagnostic. La sensibilité diagnostique du scanner couplé à une myélographie est théoriquement comparable à celle de l’IRM, mais le risque de dissémination septique intraméningé contre-indique formellement cette technique en cas de suspicion de localisation lombaire. Les autres désavantages du myéloscanner sont l’injection intra-thécale d’un produit de contraste iodé et l’irradiation. L’extension de l’AES et le degré de sténose du canal rachidien sont des éléments pronostics reconnus [4]. Au début du XXe siècle, avant l’ère des antibiotiques, la mortalité était supérieure à 80%. Elle est actuellement de 5-15%. L’AES est une urgence neurochirurgicale et requiert un traitement immédiat. L’apparition d’un déficit neurologique est de mauvais pronostic. Une paraplégie évoluant depuis plus de 36 heures est généralement irréversible. C’est essentiellement l’effet mécanique compressif qui est à l’origine des déficits neurologiques et non pas d’hypothétiques thromboses vasculaires septiques. Le traitement est le drainage chirurgical nécessitant le plus souvent une laminectomie pluriétagée, associé à une antibiothérapie de 4-6 semaines. Le risque de dissémination transméningée avec pour complication une méningite voire une myélite septique est réel.

## Conclusion

L’AES est une pathologie rare mais non exceptionnelle. Il complique le plus souvent une infection locorégionale ou une procédure interventionnelle sur le rachis. La présence d’un AES isolé, sans facteur de risque, est une éventualité rare mais à laquelle il faut penser devant toute rachialgie fébrile accompagnée d’un syndrome inflammatoire biologique. L’introduction précoce d’un drainage chirurgical associé à une antibiothérapie a nettement amélioré le pronostic en évitant l’apparition de séquelles neurologiques irréversibles. La sensibilité de l’IRM en fait l’examen de référence. L’identification du germe est l’étape diagnostique indispensable pour cibler l’antibiothérapie.
